# Meta‐analysis of the impact of neoadjuvant therapy on patterns of recurrence in pancreatic ductal adenocarcinoma

**DOI:** 10.1002/bjs5.46

**Published:** 2018-03-30

**Authors:** S. Schorn, I. E. Demir, N. Samm, F. Scheufele, L. Calavrezos, M. Sargut, R. M. Schirren, H. Friess, G. O. Ceyhan

**Affiliations:** ^1^ Department of Surgery, Klinikum rechts der Isar, School of Medicine Technical University of Munich, Ismaningerstrasse 22 D‐81675 Munich Germany

## Abstract

**Background:**

Neoadjuvant therapy may increase the rate of radical tumour resection in patients with pancreatic cancer. Its impact on tumour recurrence has not been investigated fully. This study aimed to assess the impact of neoadjuvant therapy on patterns of recurrence.

**Methods:**

A systematic review was performed of articles identified through the PubMed, Scopus, Embase, Ovid and Google Scholar databases that analysed the relationship between neoadjuvant therapy and recurrence published to January 2016. The main endpoint was overall tumour recurrence. Other endpoints included local recurrence, any kind of distant, hepatic, pulmonary or peritoneal metastasis.

**Results:**

A total of 4257 citations were reviewed. Twelve observational studies comprising 1365 patients were analysed. Neoadjuvant therapy significantly reduced the risk of overall (risk ratio (RR) 0·82, 95 per cent c.i. 0·74 to 0·90; P < 0·001) and local (RR 0·42, 0·32 to 0·55; P < 0·001) recurrence. Neoadjuvant therapy did not reduce the risk of any kind of distant (RR 1·02, 0·91 to 1·14; P = 0·78), hepatic (RR 0·86, 0·68 to 1·10; P = 0·23), pulmonary (RR 0·99, 0·37 to 2·66; P = 0·98) or peritoneal (RR 0·88, 0·57 to 1·38; P = 0·58) metastasis.

**Conclusion:**

Neoadjuvant therapy reduced the risk of local recurrence but not that of distant metastasis.

## Introduction

The prognosis of patients with pancreatic ductal adenocarcinoma (PDAC) is dismal, with a 5‐year overall survival rate of approximately 5 per cent^1^. Rates of curative resection remain low and adjuvant therapy failure, even after curative resection, has only limited impact^2^, ^3^, ^4^, ^5^, ^6^. Neoadjuvant therapy has been advocated in an attempt to increase radical resections, decrease distant recurrence and improve selection of patients for surgery.

Several studies have investigated the impact of neoadjuvant treatment in resectable (RPC), borderline resectable (BRPC) and locally advanced (LAPC) pancreatic cancer^7^. Although higher postoperative morbidity and mortality rates were observed in the neoadjuvant therapy group, patients with BRPC or LAPC who successfully underwent resection after this treatment had an increased median survival time of 20·5 months, compared with 10·2 months in patients with unresectable disease after neoadjuvant therapy, or 6–11 months in patients who received only palliative treatment. No effect of neoadjuvant therapy on overall survival was observed in patients with RPC who had adjuvant therapy. A study^8^ comparing the outcome of patients with BRPC found 1‐ and 2‐year survival rates of 80·0 and 65·2 per cent respectively in those who had neoadjuvant treatment *versus* 66·7 and 16·0 per cent in those who had no neoaduvant therapy. A further study^9^ reported that neoadjuvant therapy improved the 2‐year survival rate (59·7 per cent, compared with 47·6 per cent with no neoadjuvant therapy) and the 5‐year survival rate (26·1 *versus* 18·9 per cent respectively), and decreased 2‐year (23·3 *versus* 42·6 per cent) and 5‐year (29·7 *versus* 45·9 per cent) local recurrence rates in patients with primary RPC.

No in‐depth meta‐analysis has been performed to compare the true impact of neoadjuvant therapy *versus* upfront surgery on tumour recurrence in PDAC. In this study, a systematic review and meta‐analysis was performed of studies comparing patterns of recurrence in patients with RPC, BRPC and LAPC who either received neoadjuvant therapy or underwent primary surgery.

## Methods

### Search strategy

The decision was made to follow the PRISMA guidelines^10^ as they can be used for observational studies and are widely accepted to increase the quality of systematic reviews^11^, ^12^, ^13^. Moreover, each included study was screened for 34 STROBE items^14^, and percentages from 0 (indicating the worst study design) to 100 (expressing a perfectly designed study) were calculated. For the systematic review, PubMed, Scopus, Embase, Ovid and Google Scholar databases were screened systematically. Search terms included ‘recurrence’, ‘pancreatic cancer’, ‘pancreatic ductal adenocarcinoma’, ‘neoadjuvant therapy’, ‘preoperative therapy’, ‘neoadjuvant chemotherapy’ and ‘neoadjuvant chemoradiotherapy’. All studies with a comparison of surgical treatment with and without neoadjuvant therapy published to January 2016 were included.

### Data extraction

To minimize selection bias, two reviewers independently screened topics, abstracts and full‐text articles for the systematic review. Duplicates, all publications without clinical data, reviews and articles published in languages other than English were excluded. All articles that did not specifically identify PDAC or did not provide a comparison of recurrence rates after neoadjuvant therapy *versus* primary surgery were excluded. Only studies containing numerical data regarding recurrent disease after curative surgery with or without previous chemo(radio)therapy were included. Systematic reviews and meta‐analyses were not considered, but were used to find additional studies.

Full‐text articles were analysed, and data extracted to correlate the effect of neoadjuvant therapy on pattern of tumour recurrence. The primary endpoint of the meta‐analysis was the overall incidence of tumour recurrence after curative resection. Secondary endpoints included local recurrence, distant metastasis, hepatic metastasis, pulmonary metastasis and peritoneal metastasis. Patients with histologically proven PDAC were included. Other solid malignancies arising from or involving the pancreas (such as ampullary cancer, distal cholangiocarcinoma, adenosquamous cancers and neuroendocrine tumours) were excluded. Studies that contained patients who all received neoadjuvant or adjuvant therapy, single‐arm phase I/II trials, and all other studies comparing two different neoadjuvant models that did not include an arm with primary surgery patients, were also excluded. All studies in which no curative operation could be achieved after neoadjuvant therapy were excluded. In this context, studies with bypass operations after neoadjuvant therapy or those including a large proportion of patients undergoing R2 resection were excluded from further analysis.

To minimize the risk of including doubled patients' data, only one study per study group was included in the meta‐analyses. When there were multiple publications by a group, data were presented to an independent reviewer, who decided to include the most recent paper or the study that had accrued most patients.

### Statistical analysis

RevMan™, version 5.3 software (The Nordic Cochrane Centre, The Cochrane Collaboration, Copenhagen, Denmark) was used for meta‐analysis. The exact numbers of patients with tumour recurrence, local recurrence, or any kind of distant, hepatic, pulmonary or peritoneal metastasis were pooled. Heterogeneity between the included studies of the different meta‐analysis was quantified using the inconsistency statistic (*I* ^2^). When heterogeneity was absent (*I* ^2^ = 0) or low level (*I* ^2^ less than 50 per cent) in the meta‐analysis, the Mantel–Haenszel fixed‐effect method was used to pool data. When *I* ^2^ exceeded 50 per cent, a high level of heterogeneity was assumed and the Mantel–Haenszel random‐effects method was used, providing an estimate for an average risk ratio (RR). All results of meta‐analyses are expressed as the pooled RR with 95 per cent confidence intervals. A two‐sided *P* value was calculated, and a level of significance of α = 0·05 was used. Results of the STROBE items are expressed as median (range) values.

To investigate publication bias, funnel plots were made using RevMan™ version 5.3. According to the recommendations of the Cochrane Collaboration^15^, funnel plots were performed only for meta‐analyses including more than ten studies. To ensure the absence of publication bias, Egger's regression test was used (StatsDirect® version 3.0.167 software; StatsDirect, Altrincham, UK).

To provide insight into the risk of tumour recurrence after neoadjuvant therapy, subgroup analyses were performed. Patients were divided into two subgroups. The RPC subgroup included studies in which patients were staged as having resectable disease and either received neoadjuvant therapy followed by curative tumour resection or underwent surgery first. Data from all other studies included patients with heterogeneous tumour stages before neoadjuvant therapy or surgery (RPC, BRPC and LAPC); these were pooled in a second subgroup analysis. The overall pooled effect of both subgroups was estimated, providing an adequate RR for tumour recurrence pattern.

In addition, to provide a more accurate insight into the risk of overall tumour recurrence after neoadjuvant therapy, subgroup analyses were performed including only studies in which most patients received any kind of neoadjuvant radio(chemo)therapy. Consequently, studies that only included patients after neoadjuvant chemotherapy were excluded for this subgroup analysis.

## Results

### Search results and characteristics of included studies

**Figure 1:**
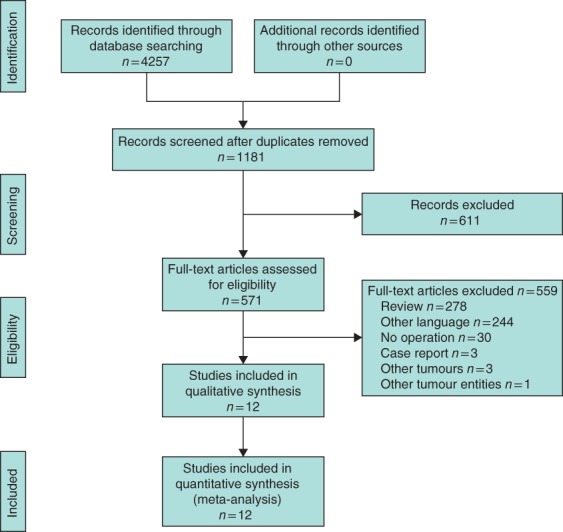
PRISMA flow diagram for the systematic review and meta‐analysis

Of 4257 citations identified, 12 studies were included in the meta‐analyses (*Fig*. 1 and *Table* 1). All studies that were eligible for meta‐analysis were observational. Patients in the primary surgery group were more often classified as having resectable disease (672 of 733, 91·7 per cent), with a minority having BRPC (59 patients, 8·0 per cent) or LAPC (2 patients, 0·3 per cent). In contrast, 212 (33·5 per cent) of the 632 patients in the neoadjuvant therapy group were classified as having BRPC and 46 (7·3 per cent) as having LAPC before receiving neoadjuvant treatment.

**Table 1:** Characteristics of studies included in systematic review and meta‐analysis

Reference	Type of tumour		Neoadjuvant chemotherapy	Neoadjuvant CRT	Conclusion	STROBE (%)
	Neoadjuvant therapy	Primary surgery				
Barbier et al. 6	RPC 35	RPC 64	–	5‐FU/cisplatin CRT	Neoadjuvant therapy improved local tumour control with no benefit on OS and distant metastasis	66·7
Ferrone et al. 16	RPC 12, BRPC 9, LAPC 19	RPC 87	FOLFIRINOX: 16	FOLFIRINOX + 50·4 Gy: 24	Neoadjuvant therapy improved OS	56·7
Fujii et al. 8	BRPC 18	BRPC 50	–	S‐1, tegafur (5‐FU) with oteracil or gimeracil CRT (50·4 Gy)	In patients with BRPC, chemoradiotherapy followed by surgery increased the incidence of N0 and R0 resection with increased OS	60·0
Greer et al. 17	RPC 16, BRPC 20, LAPC 6	RPC 47, BRPC 9, LAPC 1	–	5‐FU/cisplatin, gemcitabine‐based chemotherapy (45–50·4 Gy)	Neoadjuvant therapy improved local tumour control with no effect on OS	43·3.
Jiang et al. 18	RPC 112	RPC 120	–	5‐FU or gemcitabine‐based CRT (46–50 Gy)	Neoadjuvant therapy improved local tumour control and improved OS	63·3
Katz et al. 19	RPC 106, BRPC 41	RPC 46, LAPC 1	–	Gemcitabine, 5‐FU or capecitabine CRT (30 or 50·4 Gy) + neoadjuvant gemcitabine‐based chemotherapy	Preoperative chemoradiotherapy and meticulous dissection of the SMA enlarges the distance between tumour cells and SMA and may therefore influence local tumour control	66·7
Massucco et al. 20	BRPC 18, LAPC 10	RPC 44	–	Gemcitabine CRT (45 Gy)	Patients with BRPC and neoadjuvant therapy followed by curative surgery had at least the same survival as patients with RPC and upfront surgery	56·5
Moutardier et al. 21	RPC 23	RPC 17	–	5‐FU/cisplatin CRT (30–40 Gy)	Neoadjuvant therapy increased OS	73·3
Papavasiliou et al. 22	BRPC 106	RPC 166	–	5‐FU or gemcitabine‐based CRT (52 Gy)	Neoadjuvant therapy improved disease‐free survival and OS, and decreased local recurrence and hepatic metastasis rates	66·7
Satoi et al. 23	RPC 16, LAPC 11	RPC 41	–	Cisplatin, 5‐FU or gemcitabine CRT (40 Gy)	Neoadjuvant therapy increased resectability rates and improved OS in curative cases	66·7
Spitz et al. 24	RPC 41	RPC 19	–	5‐FU CRT (30 or 50·4 Gy)	Neoadjuvant therapy followed by curative surgery did not provide any benefit for tumour recurrence and OS	63·3
Tajima et al. 25	RPC 13	RPC 21	Gemcitabine + S‐1		Neoadjuvant therapy did not affect 1‐ and 3‐year survival rates and had no impact on tumour recurrence	60·0

CRT, chemoradiotherapy; RPC, resectable pancreatic cancer; 5‐FU, 5‐fluorouracil; OS, overall survival; BRPC, borderline resectable pancreatic cancer; LAPC, locally advanced pancreatic cancer; FOLFIRINOX, leucovorin (folinic acid), fluorouracil, irinotecan and oxaliplatin.

The most important features of the individual studies that were included in the systematic review and meta‐analysis are summarized in Table 1 6 8, 16, 17, 18, 19, 20, 21, 22, 23, 24, 25. The majority of studies^6^ ^8^, ^16^, ^17^, ^18^, ^19^, ^20^, ^21^, ^22^, ^23^, ^24^ compared neoadjuvant chemoradiotherapy with primary surgery. Only a single study^25^ compared neoadjuvant chemotherapy with primary surgery, and another^16^ included both neoadjuvant regimens in the analyses. Definitions of resectability rates varied between the studies. Some defined resectability rates as ‘true’ intention‐to‐treat rates starting from the number of patients before neoadjuvant therapy to curative resection, whereas other studies only included patients who showed regressive or stable disease on imaging after neoadjuvant therapy and who underwent surgery. Three studies^6^ ^8^, ^24^ provided dropout rates of patients in the primary surgery group who were understaged at primary presentation and were not able to undergo curative surgery. Regarding this heterogeneity in the definition of resectability rates and the lack of information in the upfront surgery group, no reliable information could be extracted regarding dropout rates of patients between the neoadjuvant therapy and primary surgery group. In addition, to assess the quality of included studies, the 34 STROBE items were used; the median percentage was 63·3 (range 43·3–73·3) per cent.

### Overall recurrence

**Figure 2:**
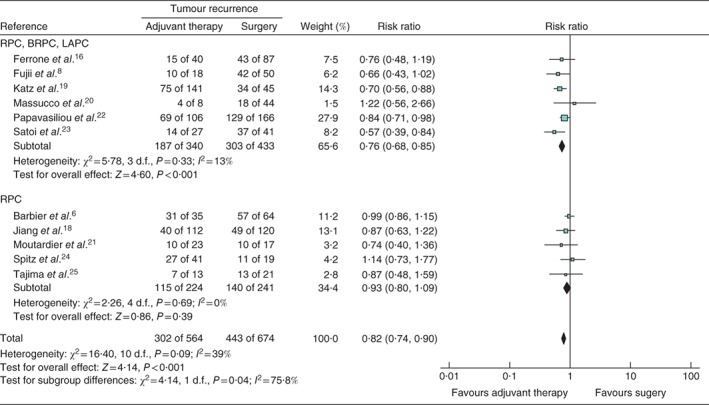
Forest plot comparing overall tumour recurrence following the use of neoadjuvant therapy versus primary surgery in patients with pancreatic cancer. A Mantel–Haenszel fixed‐effect model was used for meta‐analysis. Risk ratios are shown with 95 per cent confidence intervals. RPC, resectable pancreatic cancer; BRPC, borderline resectable pancreatic cancer; LAPC, locally advanced pancreatic cancer

The data of patients who had any kind of tumour recurrence following curative resection of PDAC were included. The pooled estimated RR of 11 studies^6^ ^8^, ^16^ ^18^, ^19^, ^20^, ^21^, ^22^, ^23^, ^24^, ^25^ demonstrated that the risk of developing any recurrence was lower in patients who received neoadjuvant therapy (RR 0·82, 95 per cent c.i. 0·74 to 0·90; P < 0·001) (Fig. 2 a). Only a low level of heterogeneity was present in this meta‐analysis (I ^2^ = 39 per cent), so the fixed‐effect model was used to perform a meta‐analysis that included 564 patients in the neoadjuvant therapy group and 674 in the primary surgery group. Recurrences were observed in 53·5 and 65·7 per cent of the patients respectively. Duration of follow‐up ranged from 11 months^16^ to 30 months^25^, with an overall median of 18·5 months.

The subgroup analyses indicated that, among patients with RPC, there was no statistically significant change in the risk of developing tumour recurrence after neoadjuvant treatment (RR 0·93, 95 per cent c.i. 0·80 to 1·09; *P* = 0·39) (*Fig*. 2 *a*). In the subgroup of studies that included more advanced tumour stages, patients with RPC, BRPC or LAPC who received neoadjuvant therapy had a decreased risk of recurrence compared with patients who had surgery first (RR 0·76, 0·68 to 0·85; *P* < 0·001) (*Fig*. 2 *a*).

### Site of recurrence

**Figure 3:**
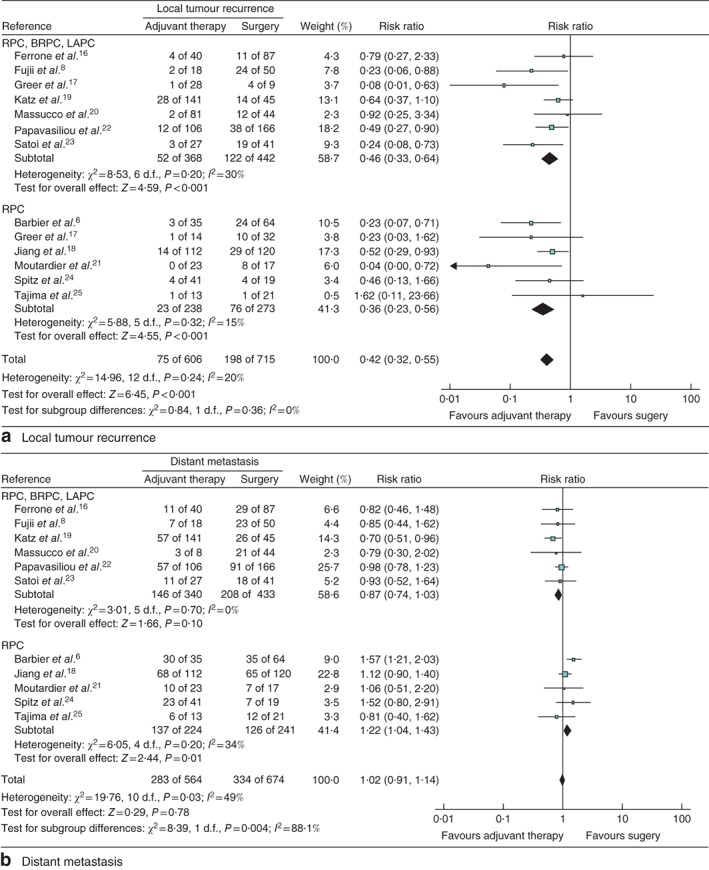
Forest plot comparing **a** local tumour recurrence and **b** distant metastasis of any kind following the use of neoadjuvant therapy versus primary surgery in patients with pancreatic cancer. A Mantel–Haenszel fixed‐effect model was used for meta‐analysis. Risk ratios are shown with 95 per cent confidence intervals. RPC, resectable pancreatic cancer; BRPC, borderline resectable pancreatic cancer; LAPC, locally advanced pancreatic cancer

To analyse the impact of neoadjuvant therapy on local tumour recurrence, 12 studies^6^ ^8^, ^16^, ^17^, ^18^, ^19^, ^20^, ^21^, ^22^, ^23^, ^24^, ^25^ were identified. Of those undergoing primary surgery, 27·7 per cent developed local recurrence compared with 12·4 per cent in the neoadjuvant group (*Fig*. 3 *a*). The estimated heterogeneity between studies was low. In contrast to local recurrence, neoadjuvant therapy did not influence distant recurrence, which developed in 283 (50·2 per cent) of 564 patients in the neoadjuvant group *versus* 334 (49·6 per cent) of 674 in the surgery‐first group. The estimated RR of the meta‐analysis, which included 11 studies^6^ ^8^, ^16^ ^18^, ^19^, ^20^, ^21^, ^22^, ^23^, ^24^, ^25^, also failed to show any relevant impact of neoadjuvant therapy on the risk of distant metastasis (RR 1·02, 95 per cent c.i. 0·91 to 1·14; *P* = 0·78) (*Fig*. 3 *b*).

This effect was more pronounced in the subgroup analysis, in which patients with resectable disease showed a decreased risk of developing local recurrence. Distant metastasis in patients with RPC after neoadjuvant therapy was associated with an increased risk of developing distant recurrence compared with that in the primary surgery group (RR 1·22, 1·04 to 1·43; *P* = 0·01) (*Fig*. 3 *b*). No effect was seen in the subgroup of patients with RPC, BRPC or LAPC (RR 0·87, 0·74 to 1·03; *P* = 0·10) (*Fig*. 3 *b*).

### Distant and peritoneal metastases

The overall risk of developing hepatic metastasis was not affected by neoadjuvant therapy: recurrence rate 27·3 per cent *versus* 29·4 per cent for primary surgery. The subgroup analysis revealed a beneficial effect of neoadjuvant treatment in patients with BRPC and LAPC, but not in those with RPC (*Fig. S1a*, supporting information).

The risk of pulmonary (RR 0·99, 95 per cent c.i. 0·37 to 2·66; *P* = 0·98) (*Fig. S1b*, supporting information) and peritoneal (RR 0·88, 0·57 to 1·38; *P* = 0·58) (*Fig. S1c*, supporting information) recurrence was no different between neoadjuvant therapy and primary surgery groups.

### Type of neoadjuvant therapy

Neoadjuvant chemoradiotherapy reduced the risk of any kind of tumour recurrence (RR 0·82, 95 per cent c.i. 0·75 to 0·91; *P* < 0·001) (*Fig. S2a*, supporting information), predominantly by reducing local recurrence (RR 0·40, 0·30 to 0·52; *P* < 0·001) (*Fig. S2b*, supporting information). No statistically significant effect was observed on the overall risk of distant metastasis (RR 1·04, 0·92 to 1·17; *P* = 0·53) (*Fig. S2c*, supporting information), hepatic metastasis (RR 0·92, 0·72 to 1·19; *P* = 0·55) (*Fig. S3a*, supporting information), pulmonary metastasis (RR 0·82, 0·27 to 2·44; *P* = 0·72) (*Fig. S3b*, supporting information) or peritoneal recurrence (RR 0·89, 0·55 to 1·44; *P* = 0·63) (*Fig. S3c*, supporting information).

### Risk of publication bias in the meta‐analyses

**Figure 4:**
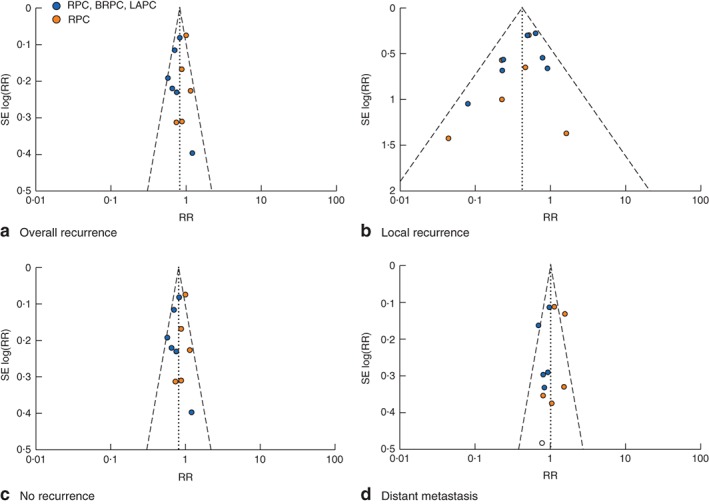
Funnel plot of publication bias in meta‐analyses of **a** overall recurrence (11 studies), **b** local recurrence (11 studies), **c** no recurrence (10 studies) and **d** distant metastasis (11 studies). RR, risk ratio; RPC, resectable pancreatic cancer; BRPC, borderline resectable pancreatic cancer; LAPC, locally advanced pancreatic cancer

Funnel plots for the meta‐analyses of overall recurrence, local recurrence, no recurrence and distant metastasis using the fixed‐effect method to pool RRs are shown in *Fig*. 4 *a–d*. All plots displayed a nearly equivalent distribution of the individual studies, demonstrating no publication bias. Egger's regression test confirmed the absence of significant publication bias.

## Discussion

Neoadjuvant therapy provided more efficient local tumour control than primary surgery, but did not appear to influence the development of peritoneal and distant metastases. As ten of the 12 studies used neoadjuvant chemoradiotherapy and only two^16^ ^25^ included patients receiving neoadjuvant chemotherapy, local effects of radiotherapy on recurrence may be a factor.

Several systematic reviews with meta‐analysis have been performed to analyse the effect of neoadjuvant therapy in patients with PDAC on survival^26^, ^27^, ^28^, R0 resection rates^26^ and resectability rates^28^. Although the clinical benefit of neoadjuvant therapy on overall and progression‐ and disease‐free survival is still debated, its effect on relevant histopathological features of PDAC is well understood^22^ ^29^. Some studies^22^ ^29^ have shown that neoadjuvant therapy can decrease tumour size, increase the rate of tumour‐free lymph nodes, and improve the rate of tumour‐free resection margins. The effect of neoadjuvant therapy on preoperative primary tumour classification as RPC or BRPC revealed, for both groups, that patients receiving this treatment had smaller tumours, better UICC stage and more tumour‐free lymph nodes than patients who had primary surgery^29^. In a recent meta‐analysis^30^, neoadjuvant therapy reduced not only tumour size, tumour‐positive lymph nodes and the rate of positive resection margins, but also the rate of dedifferentiated cancer and lymphatic vessel invasion.

The use of preoperative chemotherapy alone (FOLFIRINOX – leucovorin (folinic acid), fluorouracil, irinotecan and oxaliplatin) was associated with less frequent perineural invasion and fewer tumour positive lymph nodes compared with primary surgery^16^. Although the majority of patients were still diagnosed radiologically as having unresectable disease, a higher rate of tumour‐free resection margins could be achieved than with resection alone^16^. Despite a lower frequency of RPC in the group receiving neoadjuvant chemotherapy (30 *versus* 100 per cent in the primary surgery group), a clinically relevant increase in overall survival was observed. High rates of R0 resection and the more favourable postoperative outcome were in contrast to the preoperative radiological assessment, suggesting that this is unreliable in patients who have received neoadjuvant treatment.

In contrast to neoadjuvant therapy, adjuvant chemotherapy for patients following curative resection is undebated. In the ESPAC‐1 trial, Neoptolemos and colleagues^31^ compared overall survival of patients with and without adjuvant chemotherapy after curative resection. Patients who received adjuvant chemotherapy had an increased median survival of 20·1 months, compared with 15·5 months in those who did not (hazard ratio (HR) 0·71, 95 per cent c.i. 0·55 to 0·92; *P* = 0·009). Recently, the results of the ESPAC‐4 study^32^ comparing gemcitabine alone with gemcitabine plus capecitabine demonstrated that median survival was improved with the combination (28·0 *versus* 25·5 months for gemcitabine alone; HR 0·82, 0·68 to 0·98, *P* = 0·032). Data from the ESPAC trials underline the benefit of adjuvant chemotherapy in patients with pancreatic cancer.

The major limitation of the present meta‐analysis is that all included studies were observational. No completed RCT has compared the effects of neoadjuvant chemotherapy or neoadjuvant chemoradiotherapy in PDAC. Thus bias, including selection bias, is present, as definitions of RPC, BRPC and LAPC varied, as did indications for administering neoadjuvant therapy. The type and timing of follow‐up after treatment differed considerably between institutions and clinicians. Compared with mortality, recurrence may be open to interpretation, as the disease may recur clinically, biochemically, radiologically or pathologically. The site of recurrence may be even misinterpreted more frequently, as there is no clinical need to rule out peritoneal metastases if liver metastases have been detected radiologically. For this purpose, included studies were screened for information on follow‐up; all but two studies^17^ ^21^ provided sufficient information. Follow‐up included CT of the abdomen every 3–6 months in six studies^19^ ^21^, ^22^, ^23^, ^24^, ^25^, and ultrasound examination every 4 months and CT after 12 months or on demand in two studies^18^ ^20^. Another study^6^ performed CT at 1, 4, 6, and then every 12 months after surgery. Only two studies^8^ ^17^ did not include information on their follow‐up examinations. As well as the quality of follow‐up data, STROBE items were used to provide an objective statement on the included studies. The median percentage of fulfilled STROBE criteria was 63·3 (range 43·3–73·3) per cent. The STROBE criteria suggest that the methodical quality of the studies was comparable.

Studies were not clear about the numbers of dropouts during neoadjuvant treatment, owing to progressive disease or unresectable tumours at surgical exploration. The different effects of neoadjuvant therapy in patients with RPC, BRPC and LAPC should be stratified according to their radiological tumour stage to avoid any bias caused by mixed cohorts. Only well designed trials will further clarify the true effect of neoadjuvant therapy in pancreatic cancer.

## Disclosure

The authors declare no conflict of interest.

## Supporting information


**Fig. S1** Forest plot comparing **a** hepatic, **b** pulmonary and **c** peritoneal metastasis following the use of neoadjuvant therapy versus primary surgery in patients with pancreatic cancer. Mantel–Haenszel (M‐H) models were used for meta‐analysis. Risk ratios are shown with 95 per cent confidence intervals. RPC, resectable pancreatic cancer; BRPC, borderline resectable pancreatic cancer; LAPC, locally advanced pancreatic cancer
**Fig. S2** Forest plot comparing **a** tumour recurrence, **b** local recurrence and **c** distant metastasis following the use of neoadjuvant chemoradiotherapy (NCRTX) versus primary surgery in patients with pancreatic cancer. Mantel–Haenszel (M‐H) models were used for meta‐analysis. Risk ratios are shown with 95 per cent confidence intervals. RPC, resectable pancreatic cancer; BRPC, borderline resectable pancreatic cancer; LAPC, locally advanced pancreatic cancer
**Fig. S2** Forest plot comparing **a** hepatic, **b** pulmonary and **c** peritoneal metastasis following the use of neoadjuvant chemoradiotherapy (NCRTX) versus primary surgery in patients with pancreatic cancer. Mantel–Haenszel (M‐H) models were used for meta‐analysis. Risk ratios are shown with 95 per cent confidence intervals. RPC, resectable pancreatic cancer; BRPC, borderline resectable pancreatic cancer; LAPC, locally advanced pancreatic cancer
