# Supplementary material for: Meta‐analysis of the impact of neoadjuvant therapy on patterns of recurrence in pancreatic ductal adenocarcinoma
Source: BJS Open. 2018 Mar 30;2(2):52–61. doi: 10.1002/bjs5.46 (PMC5989995; doi:10.1002/bjs5.46)
Supplement: Supplementary file 1 — Fig. S1 Forest plot comparing a hepatic, b pulmonary and c peritoneal metastasis following the use of neoadjuvant therapy versus primary surgery in patients with pancreatic cancer. Mantel–Haenszel (M‐H) models were used for meta‐analysis. Risk ratios are shown with 95 per cent confidence intervals. RPC, resectable pancreatic cancer; BRPC, borderline resectable pancreatic cancer; LAPC, locally advanced pancreatic cancer Fig. S2 Forest plot comparing a tumour recurrence, b local recurrence and c distant metastasis following the use of neoadjuvant chemoradiotherapy (NCRTX) versus primary surgery in patients with pancreatic cancer. Mantel–Haenszel (M‐H) models were used for meta‐analysis. Risk ratios are shown with 95 per cent confidence intervals. RPC, resectable pancreatic cancer; BRPC, borderline resectable pancreatic cancer; LAPC, locally advanced pancreatic cancer Fig. S2 Forest plot comparing a hepatic, b pulmonary and c peritoneal metastasis following the use of neoadjuvant chemoradiotherapy (NCRTX) versus primary surgery in patients with pancreatic cancer. Mantel–Haenszel (M‐H) models were used for meta‐analysis. Risk ratios are shown with 95 per cent confidence intervals. RPC, resectable pancreatic cancer; BRPC, borderline resectable pancreatic cancer; LAPC, locally advanced pancreatic cancer [file BJS5-2-52-s001.docx]

**BJS5_46**

**Meta-analysis of the impact of neoadjuvant therapy on patterns of recurrence in pancreatic ductal adenocarcinoma**

**S. Schorn, I. E. Demir, N. Samm, F. Scheufele, L. Calavrezos, M. Sargut, R. M. Schirren, H. Friess and G. O. Ceyhan**


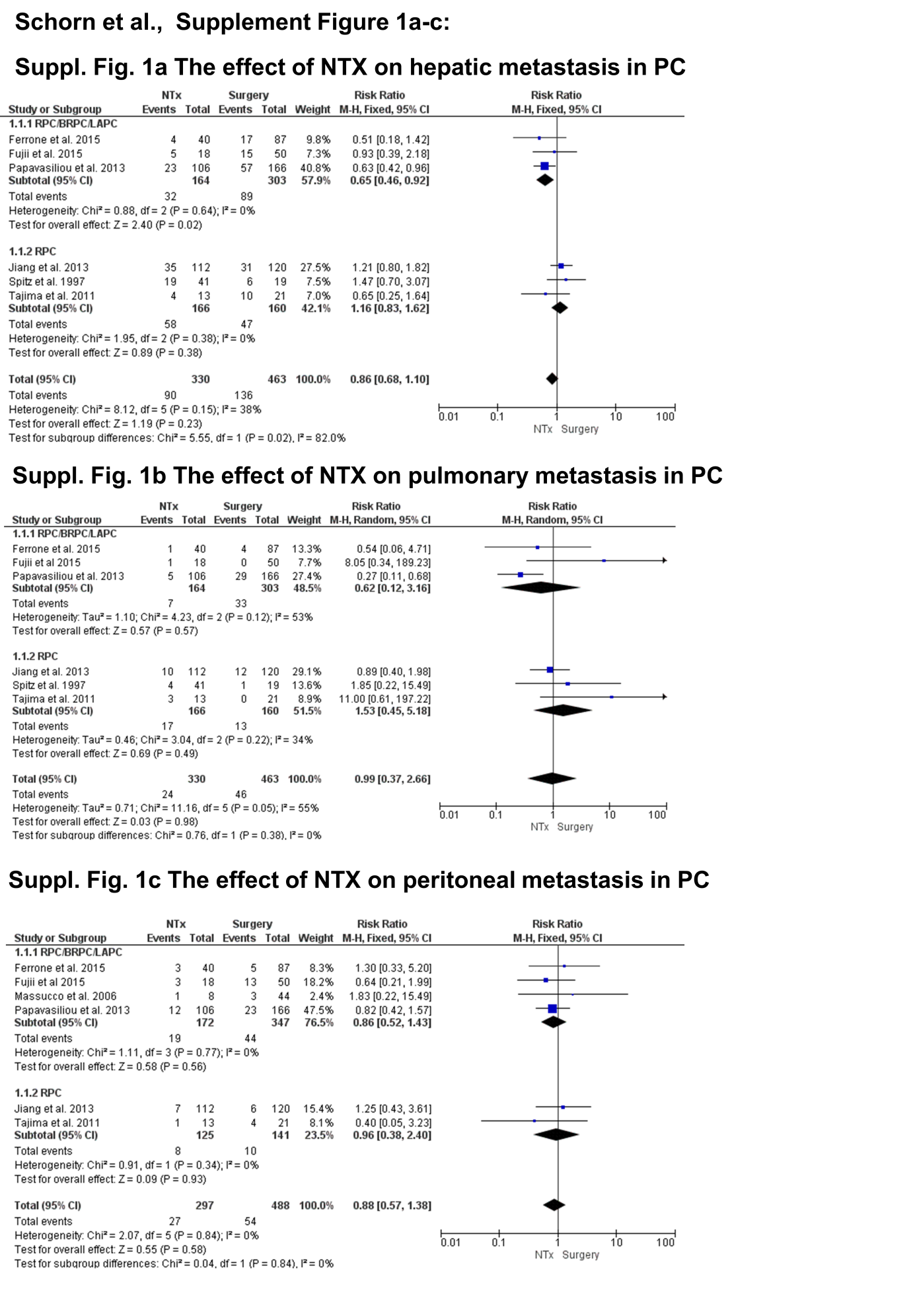
**Fig. S1** Forest plot comparing **a** hepatic, **b** pulmonary and **c** peritoneal metastasis following the use of neoadjuvant therapy *versus* primary surgery in patients with pancreatic cancer. Mantel–Haenszel (M-H) models were used for meta-analysis. Risk ratios are shown with 95 per cent confidence intervals. RPC, resectable pancreatic cancer; BRPC, borderline resectable pancreatic cancer; LAPC, locally advanced pancreatic cancer


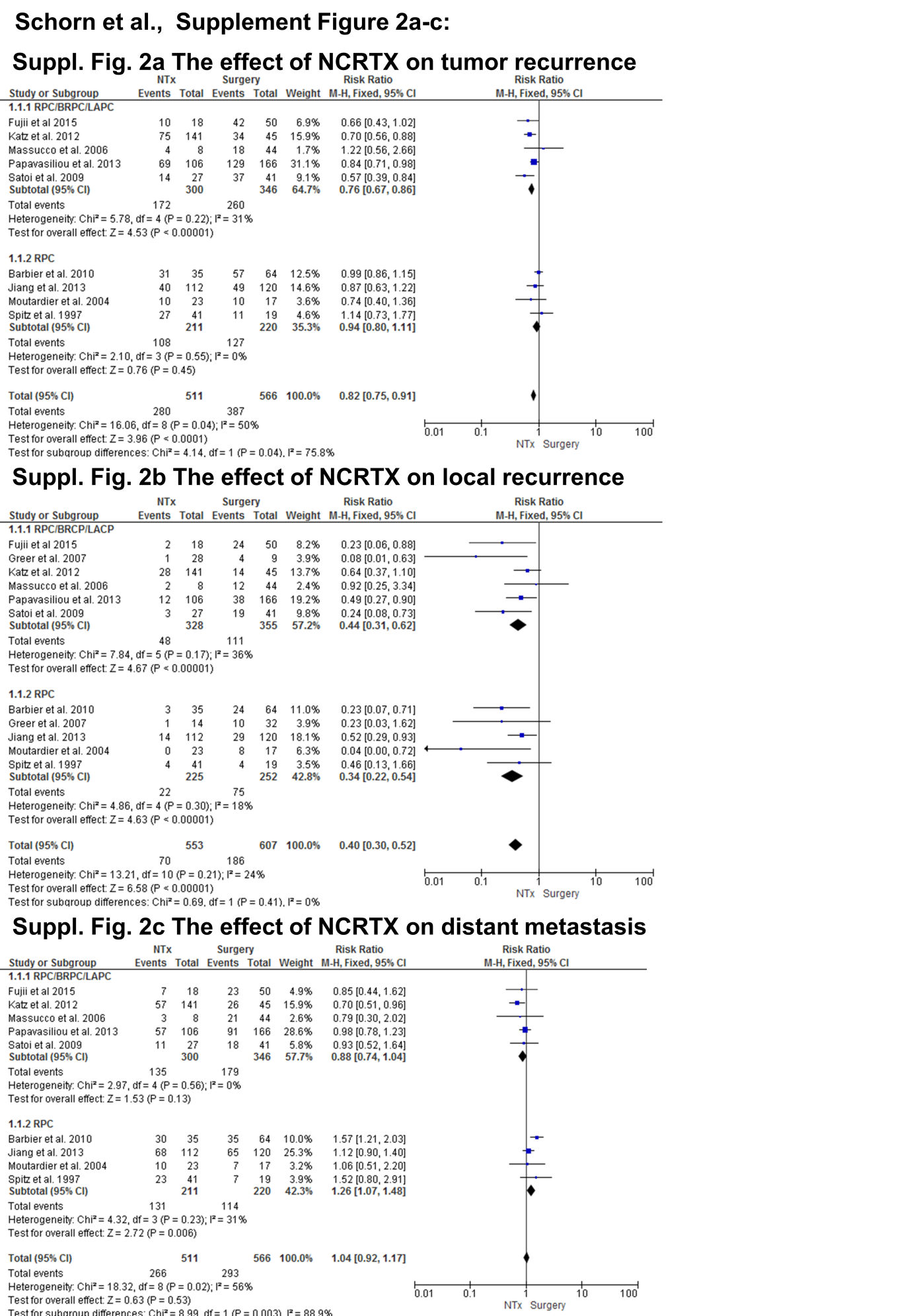


**Fig. S2** Forest plot comparing **a** tumour recurrence, **b** local recurrence and **c** distant metastasis following the use of neoadjuvant chemoradiotherapy (NCRTX) *versus* primary surgery in patients with pancreatic cancer. Mantel–Haenszel (M-H) models were used for meta-analysis. Risk ratios are shown with 95 per cent confidence intervals. RPC, resectable pancreatic cancer; BRPC, borderline resectable pancreatic cancer; LAPC, locally advanced pancreatic cancer


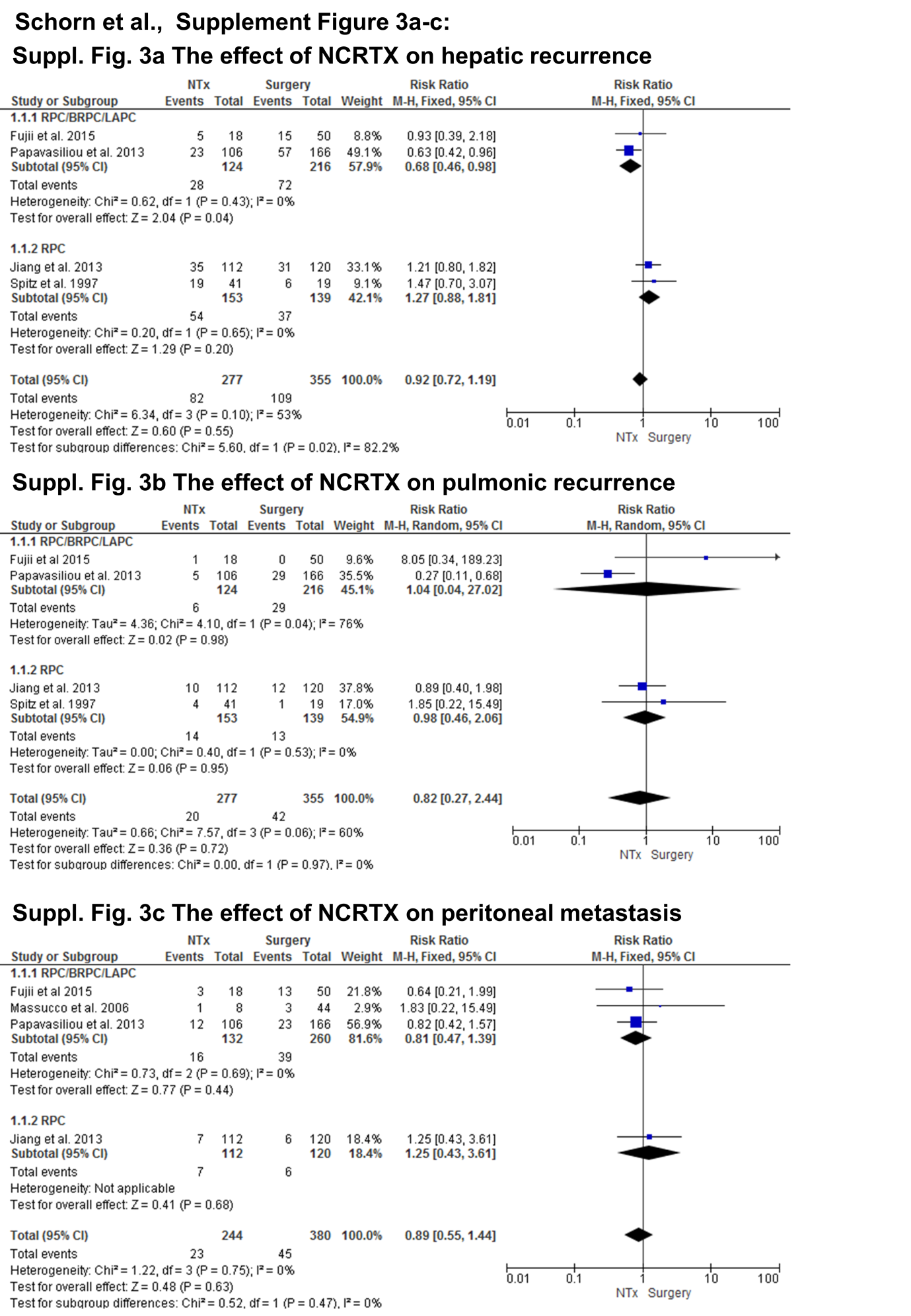


**Fig. S2** Forest plot comparing **a** hepatic, **b** pulmonary and **c** peritoneal metastasis following the use of neoadjuvant chemoradiotherapy (NCRTX) *versus* primary surgery in patients with pancreatic cancer. Mantel–Haenszel (M-H) models were used for meta-analysis. Risk ratios are shown with 95 per cent confidence intervals. RPC, resectable pancreatic cancer; BRPC, borderline resectable pancreatic cancer; LAPC, locally advanced pancreatic cancer
